# Influenza-associated thrombotic microangiopathies

**DOI:** 10.1007/s00467-017-3783-4

**Published:** 2017-09-07

**Authors:** Martin Bitzan, Jakub Zieg

**Affiliations:** 1 Division of Nephrology, The Montreal Children’s Hospital, McGill University Health Centre, 1001, boul. Décarie—Room B RC.6651, Montréal, QC H4A 3J1 Canada; 2 Department of Pediatric Nephrology, 2nd Faculty of Medicine, University Hospital Motol, Charles University, Prague, Czech Republic

**Keywords:** Hemolytic uremic syndrome, Thrombotic-thrombocytopenic purpura, Complement, ADAMTS13, Plasma exchange, Neuraminidase, Influenza vaccine

## Abstract

Thrombotic microangiopathy (TMA) refers to phenotypically similar disorders, including hemolytic uremic syndromes (HUS) and thrombotic thrombocytopenic purpura (TTP). This review explores the role of the influenza virus as trigger of HUS or TTP. We conducted a literature survey in PubMed and Google Scholar using HUS, TTP, TMA, and influenza as keywords, and extracted and analyzed reported epidemiological and clinical data. We identified 25 cases of influenza-associated TMA. Five additional cases were linked to influenza vaccination and analyzed separately. Influenza A was found in 83%, 10 out of 25 during the 2009 A(H1N1) pandemic. Two patients had bona fide TTP with ADAMTS13 activity <10%. Median age was 15 years (range 0.5–68 years), two thirds were male. Oligoanuria was documented in 81% and neurological involvement in 40% of patients. Serum C3 was reduced in 5 out of 14 patients (36%); Coombs test was negative in 7 out of 7 and elevated fibrin/fibrinogen degradation products were documented in 6 out of 8 patients. Pathogenic complement gene mutations were found in 7 out of 8 patients tested (C3, MCP, or MCP combined with CFB or clusterin). Twenty out of 24 patients recovered completely, but 3 died (12%). Ten of the surviving patients underwent plasma exchange (PLEX) therapy, 5 plasma infusions. Influenza-mediated HUS or TTP is rare. A sizable proportion of tested patients demonstrated mutations associated with alternative pathway of complement dysregulation that was uncovered by this infection. Further research is warranted targeting the roles of viral neuraminidase, enhanced virus-induced complement activation and/or ADAMTS13 antibodies, and rational treatment approaches.

**Electronic supplementary material:**

The online version of this article (10.1007/s00467-017-3783-4) contains supplementary material, which is available to authorized users

## Introduction

The term thrombotic microangiopathy (TMA) is used to describe a spectrum of phenotypically similar diseases characterized by intravascular (microangiopathic) hemolytic anemia (MAHA), thrombocytopenia, and in most instances, acute kidney injury (AKI). Other organ systems can be affected. Best-known examples are the hemolytic uremic syndromes (HUS) and thrombotic thrombocytopenic purpura (TTP). Although endothelial injury is a triggering event in many instances of HUS, this may not apply to the TTP and some forms of “atypical” HUS (aHUS).

Infections by Shiga toxin-producing *Escherichia coli* (STEC or Stx HUS) are the most common cause of TMA (HUS) in children. Less frequently, HUS has been linked to infections by other bacteria, such as* Shigella dysenteriae* type 1,* Clostridium perfringens* or* Streptococcus pneumoniae*, and by HIV, coxsackie-, Epstein–Barr (EBV), varicella or influenza viruses [1–3].

Thrombotic thrombocytopenic purpura, first described as a clinical entity by Moschcowitz in 1924, is now etiologically defined by the lack of plasmatic ADAMTS13 activity [4]. ADAMTS13, a metalloprotease secreted by megakaryocytes and endothelial cells, cleaves platelet-derived von Willebrand factor (VWF) “ultra large” multimers into smaller-molecular weight fragments [4, 5]. Most TTP patients have circulating anti-VWF protease antibodies, often of the IgG4 class [6]. Inherited TTP, caused by mutations in the ADAMTS13 gene, is known as Upshaw–Shulman syndrome [5, 7].

Dysregulation of the alternative pathway of complement (APC) or the coagulation system due to genetic mutations or acquired antibodies, primarily to complement factor H (CFH), increases the risk of HUS, commonly referred to as “atypical” (aHUS) [2, 8]. Genes encoding components of the coagulation and fibrinolytic cascades etiologically linked to aHUS are *THBD* (thrombomodulin), *DGKE* (diacylglycerol kinase-epsilon), *VWF* (von Willebrand factor), factor XII, and *PLG* (plasminogen) [8–12]. Other forms of “atypical” HUS (aHUS) are caused by abnormalities in the cblC pathway (methylmalonic aciduria and homocystinuria, cblC complementation type [MMACHC]) [13]. Finally, TMA may develop because of immunosuppressive and cytotoxic drugs, bone marrow transplantation, autoimmune diseases, cancer, and pregnancy. Although complement gene mutations have been identified in some of the latter conditions, the etiology is speculative in others [1, 2, 8].

This review summarizes current evidence on the link between influenza virus infection and TMA (HUS or TTP) and discusses the overlap between influenza TMA and other forms of aHUS, in addition to the diagnostic workup and management of these conditions.

## Identification of cases of influenza TMA

Using PubMed and Google Scholar, the following key words were used alone or in combination: hemolytic uremic syndrome, thrombotic thrombocytopenic purpura, thrombotic microangiopathy, influenza, influenza vaccine/vaccination. Clinical, epidemiological, and demographic features, laboratory results, treatment modalities and outcome were extracted and tabulated. English, French, and German language publications were reviewed.

## Influenza epidemiology and mechanisms of infection

Influenza viruses can cause seasonal infections and epidemics with significant morbidity and mortality. The influenza A(H1N1) pandemic in 1918–1919 was responsible for the death of an estimated 50 million people [14]. The 2009 influenza pandemic by a newly arisen influenza A(H1N1) strain caused the death of more than 280,000 persons worldwide (>12,000 in the USA) within the first year of its circulation, owing to respiratory or cardiovascular complications [15]. Seasonal influenza leads to an estimated 12,000–56,000 deaths in the USA annually [16].

Influenza virus targets the respiratory tract and causes fever, often with acute laryngitis, tracheitis, and pneumonia, and occasionally myocarditis, meningoencephalitis, or rhabdomyolysis, among other symptoms [17]. Infants and the elderly are at greatest risk of influenza-related complications. Death may occur directly by the virus or by complicating bacterial pneumonia, especially due to *S. pneumoniae*. Influenza virus belongs to the genus *Orthomyxovirus* of the *Orthomyxoviridae* family. Influenza A and B viruses contain eight antisense strand RNA segments and express at least 17 proteins, among them three membrane (glyco)proteins in the lipid envelope: hemagglutinin (HA), neuraminidase (NA), and proton channel matrix protein 2 (M2) [17]. HA and NA are genetically unstable and determine fluctuations of the prevalent subtypes of influenza virus. Viral HA mediates attachment to sialic acid-containing host cell membrane receptors and entry of the viral genome into the target cells. Human influenza strains bind terminal α2,6 galactose residues, which contributes to the known species tropism [17, 18]. Sialic acid-independent attachment has been postulated [19]. Viral neuraminidase cleaves α-ketosidic bonds of neuraminic acid [20]. It facilitates transfer of virus particles in the mucus layer of the respiratory tract and release of progeny virion from infected cells [17, 21]. NA inhibitors, such as oseltamivir (active metabolite oseltamivir carboxylate), block the release of virions and their spread to neighboring epithelial cells [21].

## Influenza-associated thrombotic microangiopathy

**Fig. 1:**
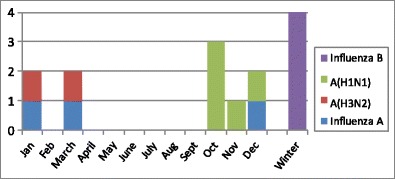
Seasonal distribution and influenza subtypes in patients with influenza-associated thrombotic microangiopathy (TMA). The occurrence of A(H1N1)-linked hemolytic uremic syndrome (HUS) coincides with the peak of the 2009 pandemic (weeks 40–51). In contrast, the expected peak of seasonal influenza A is during the first 3 months of the year [44]

Hemolytic uremic syndrome triggered by influenza virus (iHUS) is rare. In almost all instances, it is associated with influenza A virus, mainly A(H3N2) and A(H1N1). Only recently have a few cases of HUS associated with influenza B virus infection been published (Table 1) [22, 23]. Ten patients with HUS were noted during the 2009 influenza A(H1N1) pandemic [26–35], and one during a later wave [36], constituting 44% of all reported occurrences of influenza-associated thrombotic microangiopathy (iTMA; Tables 1, 2). The distribution of these cases corresponded to the course of the pandemic [44] and differed from the usual seasonal influenza pattern (Fig. 1).

**Table 1:** Distribution of influenza virus subtypes associated with thrombotic microangiopathies in humans

Influenza types^a^	Influenza A	Influenza B	References
A(H3N2)	2		[24, 25]
A(H1N1)	11		[26–36]
A (not or partially specified)	7		[37–42]
B (Yamagata)		4	[22, 23]

^a^Viral typing was omitted in one reported case [43]

**Table 2:** Clinical and laboratory characteristics of influenza thrombotic microangiopathy (iTMA) patients

		Frequency/median (range)			
		A(non-H1N1)^e^ (*n* = 10)	A(H1N1) (*n* = 11)	Influenza B (*n* = 4)	All (*n* = 25)
Demographics	Age (years)	27 (3–68), *n* = 10	15 (5–37), *n* = 11	9.5 (6–15), *n* = 4	15 (0.5–68), *n* = 5
	Female gender	7/10 (70%)	2/11 (18%)	0/4	9/25 (36%)
	Kidney transplant recipients	2/10 (20%)	1/11 (9%)	0/4	3/25 (12%)
Time	Days from influenza to HUS	4 (2–14), *n* = 8	5 (1–7), *n* = 8	2.5 (2–5), *n* = 4	4 (1–14), *n* = 20
CNS	CNS involvement	6/10 (60%)	3/11 (27%)	1/4 (25%)	10/25 (40%)
AKI	Serum creatinine (μM) at presentation	221 (65–318), *n* = 6	280 (24–698), *n* = 11	131 (89–362) *n* = 4	212 (24–698) *n* = 21
	Serum creatinine (μM) peak	408 (261–1,238) *n* = 6^f^	301 (132–701) *n* = 11	171 (89–362), *n* = 4	327 (89–1,238), *n* = 21
	Oligoanuria	5/6 (83%)	5/6 (83%)	3/4 (75%)	13/16 (81%)
	Duration of oligoanuria (days)	10 (5–28), *n* = 3	14 (9–15), *n* = 3	4 (1–7), *n* = 2	9.5 (1–28), *n* = 8
	Gross hematuria	4/8 (25%)	4/11 (36%)	2/4 (50%)	8/23 (35%)
	Proteinuria	5/5 (100%)	7/7 (100%)	4/4 (100%)	16/16 (100%)
	Renal replacement therapy	6/10 (60%)	6/11 (36%)	0/4	10/25 (40%)
	Duration of dialysis (days)	10 (5–33), *n* = 4	13 (2–28), *n* = 3	–	13 (2–33), *n* = 7
MAHA	Hemoglobin (g/L) at presentation	123 (65–171), *n* = 9	91 (65–132), *n* = 10	108.5 (57–130), *n* = 4	104 (57–171), *n* = 23
	Hemoglobin (g/L), nadir	65 (57–99), *n* = 6	76 (50–95), *n* = 7	NR	68 (50–99.3), *n* = 13
	Presence of schistocytes	7/7 (100%)	9/10 (90%)	4/4 (100%)	20/21 (95%)
	Platelets (× 10^9^/ L) at presentation	53 (6–168), *n* = 9	30 (5–254), *n* = 11	23.5 (20–58), *n* = 4	30 (5–254), *n* = 24
	Platelets (× 10^9^/ L) nadir	15 (6–56), *n* = 9	20.5 (5–80), *n* = 10	NR	20 (5–80), *n* = 19
	Platelet recovery ≥140 × 10^9^/L (days post-onset)	11 (6–16), *n* = 2	9 (6–23) *n* = 5	10 *n* = 1)	9.5 (6–23) *n* = 8
	LDH (U/L) at presentation	2,385 (200–3,016) *n* = 5	5,088 (180–13,188) *n* = 8	2,810 (≈1,100–5,218) *n* = 4	2,920 (180–13,188) *n* = 17
	LDH (U/L) peak	2,888 (2,316–4,485) *n* = 5	5,088 (300–13,188) *n* = 8	2,150 (2,150) *n* = 1	3,484 (300–13,188) *n* = 14
	Positive (direct) Coombs test	0/2	0/5	NR	0/7
Complement and coagulation	Low C3	1/3 (33%)	3/7 (43%)	1/4 (25%)	5/14 (36%)
	Low C4	0/2	0/6	0/3	0/11
	ADAMTS13 < 10%	2/3 (67%)^g^	0/2	0/1	2/6 (33%)
	Evidence of fibrinolysis (FDP)	3/5 (60%)	3/3 (100%)	NR	6/8 (75%)
	Genetic mutation^a^	1/1	2/3 (67%)	4/4 (100%)	7/8 (88%)
	Relapsing/recurrent HUS	2/10 (20%)	3/11 (27%)	3/4 (75%)	7/25 (28%)
Specific interventions	Plasma therapy	4/9 (44%)	10/11 (91%)^j^	2/4 (50%)	16/24 (67%)
	Plasma infusion (PI)	1/4 (25%)	4/10 (40%)	0/2	5/16 (31%)
	Number of PI	5 (5), *n* = 1	1 (1–14), *n* = 3	–	3 (1–14), *n* = 4
	Plasma exchange (PLEX)	3/4 (75%)	7/10 (70%)	2/4 (50%)	12/16 (75%)
	Number of PLEX sessions	6 (3–12), *n* = 3	13.5 (5–30), *n* = 6	6, *n* = 2	10 (3–30), *n* = 11
	Eculizumab^b, c^	0/10	1/11 (9%)	1/4 (25%)	2/25 (8%)
	Oseltamivir	2/9 (22%), both prior TTP	11/11 (100%), 2 prior to HUS	1/4 (25%), after onset of HUS	14/24 (58%), 4 prior to HUS
Outcome	Complete recovery	5/9 (56%)	11/11 (100%)	4/4 (100%)	20/24 (83%)
	CKD	1/6 (17%)^d, h^	0/11	0/4	1/21 (5%)
	Death	3/10 (30%)^i^	0/11	0/4	3/25 (12%)
	Graft loss (kidney transplant recipients)^d^	1/2 (50%)	0/1	–	1/3 (33%)

Oliguria, urine output <0.5 mL/kg/h for 6 h, and anuria, no urine output for >12 h (AKIN KDIGO 2012), have been combined in this table. Most authors do not provide detailed information concerning urine output. Proteinuria is defined as >1 g/day, or ≥1 g/L, >0.3 g/g creatinine or ≥2+ by dipstick

*CNS *central nervous system, *LDH* lactate dehydrogenase, *CKD* chronic kidney disease,* HUS* hemolytic uremic syndrome, *NR* not reported

^a^For details, see Table 4

^b^For details and individual reports see Supplementary Table S1

^c^References [23, 33]

^d^Graft loss (transplant recipient); patients with surviving grafts received plasma infusions and methylprednisolone pulse therapy (#5) [24] or eculizumab (#16) [33]

^e^Including one undefined strain (likely seasonal influenza A) [43]

^f^Not included are three dialyzed patients

^g^Two patients with bona fide TTP

^h^Deceased patients excluded

^i^Rapid deterioration and death (#3) on day of admission due to massive hemoptysis associated with hemorrhagic destruction of lung parenchyma and fibrin deposition in lung capillaries [38]; death due to aspiration pneumonia (#4) after initiation of dialysis, prednisone, PLEX, and splenectomy [42]; death due to myocardial infarction and heart failure (#9) in a patient with anti-ADAMTS13 TTP [39]

^j^One patient received plasma infusion and subsequently PLEX (#20) [36]

The first description of iHUS from 1971 is that of a 20-year-old kidney transplant recipient (#1) [37]. The patient was diagnosed with MAHA and graft failure 1–2 weeks after the onset of influenza, almost 2 years after renal transplantation. End stage renal disease (ESRD) was secondary to acute proliferative glomerulonephritis (GN) and malignant hypertension. She started hemodialysis 10 days before transplant nephrectomy. Additional acute laboratory features were cold agglutinins (with negative direct Coombs test) and transiently reduced plasma C3 concentration. A graft biopsy 5 weeks after HUS onset revealed thrombosis of small renal arteries and glomerular capillaries. The transplant was removed 8 weeks after HUS onset, followed by swift normalization of the hematological parameters. A subsequent graft from a deceased donor (DD) was tolerated well without recurrence of HUS.

A typical scenario of HUS due to influenza A(H1N1) infection is a previously healthy, 7-year-old boy with febrile pneumonitis and transient respiratory failure who developed severe AKI, profound MAHA, and thrombocytopenia associated with hypertensive encephalopathy 5 days after the onset of respiratory symptoms (#11). Coagulation profile, plasma fibrinogen, Coombs test, and C3 concentration were normal, as was MCP expression, plasma ADAMTS13 activity and serum CFB, CFH and CFI concentrations. He recovered completely after 2 weeks of peritoneal dialysis. No genetic studies of APC or coagulation factors were reported by the authors [28].

Relevant demographic, clinical and laboratory parameters of all patients identified with influenza A- and B-associated TMA are summarized in Table 2 (for details, see Supplementary Table S1). Three of the influenza A HUS patients had a kidney transplant at the time of infection, including #1. Patient #5 had been transplanted for chronic GN; allograft biopsy on day 10 of HUS revealed mesangiolysis and C3 deposition in the presence of normal serum C3 concentrations. Patient #16 had lost two previous allografts due to HUS caused by an activating C3 mutation [33].

Patients presented with hemolytic anemia that was associated with peripheral schistocytosis in all but one instance [33], and thrombocytopenia (nadir 5–80 × 10^9^ platelets/L). Peripheral platelet counts recovered after a median of 9.5 days (range 6–23 days; *n* = 8 patients; Table 2). Direct and indirect Coombs tests were negative in all 7 patients examined, but cold agglutinins were reported once (#1) [37]. Six of 8 patients (75%) tested for evidence for fibrinolysis showed elevated d-dimers and fibrin/fibrinogen degradation products (FDP), with normal fibrinogen levels (Table 2) [29, 32, 36, 38, 39].

**Fig. 2:**
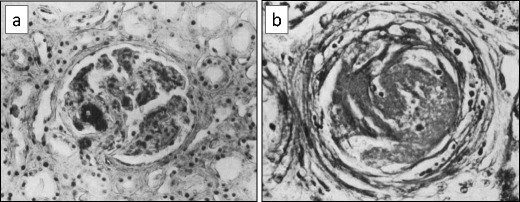
Micrographs from a patient with influenza thrombotic microangiopathy in the kidney allograft (patient #1). **a** Glomerulus with thrombosis of a capillary loop (phosphotungstic acid hematoxylin stain). **b** Cross-section of arteriole: the wall shows splitting and edema; the lumen is occluded by a thrombus (hematoxylin–eosin stain). Thrombi consisted of fibrin in addition to packed erythrocytes and thrombocytes. Some thrombi merged with the arteriolar wall, which then showed fibrinoid necrosis (reproduced from Petersen and Olsen [37], used with permission)

Acute kidney injury (AKI) developed in all 25 patients. Hypertension was present in 10 out of 23 patients (43%). Serum creatinine concentrations were already increased at first measurement in 19 out of 21 patients (90%; median 221 μM [2.4 mg/dL]) and peaked at 327 μM (3.7 mg/dL). Oliguria or anuria was documented in 13 out of 16 cases (81%). Ten patients (40%) initiated renal replacement therapy, mostly in the form of hemodialysis (median duration 13 days; Table 2). Kidney biopsies were reported in 7 patients (# 1–6, 16). An example of pertinent histopathological features of influenza A-associated HUS is shown in Fig. 2.

Central nervous system (CNS) complications, including drowsiness and mental confusion, focal neurological signs, seizures, and hemiplegia, in addition to Magnetic resonance imaging (MRI) changes and petechiae (in brain autopsy samples; Table S1) were reported in 10 iHUS patients (40%). The severity and frequency of CNS complications associated with A(H1N1) versus A(non-H1N1) influenza did not reach statistical significance (*p* = 0.20; Fisher’s exact; Table 2).

Separate analysis of the reported A(H1N1) HUS cases revealed evidence for variable abnormalities of complement and fibrinolysis, similar to the remainder of influenza A HUS cases (Table 2). It remains unclear if A(H1N1) has a greater propensity to induce HUS than other influenza subtypes [45]. Considering that there is a total disease burden of 200 million people globally [46], the proportion of (reported) HUS cases is about 0.05 per 1 million influenza A(H1N1) infections, this corresponds to two cases (# 5 and 16) among 50 million patients reported in the USA [46, 47].

The reported clinical and laboratory features of the children with influenza B-associated HUS [22, 23] resemble those described for influenza A. Interestingly, HUS was linked in all instances to genetic complement abnormalities (see below and Table 2).

## Influenza and TTP

Influenza A virus, including A(H1N1) has been invoked as a cause of TTP in at least four published reports [34, 35, 39, 40]. ADAMTS13 activity was depleted in 2 patients; both demonstrated increased anti-ADAMTS13 antibody concentrations [39, 40]. The TTP diagnosis of the remaining 2 patients was clinical, based on the combination of MAHA and neurological manifestations, while ADAMTS13 and complement studies were lacking (Table 3) [34, 35]. The mechanism leading to the rise of anti-ADAMTS13 and other autoantibodies by influenza and influenza vaccines [48, 49] warrants additional research.

**Table 3:** Demographic and clinical details of influenza-associated HUS and TTP

Features		HUS		TTP
		Undefined HUS	Genetic complement dysregulation ^c^	ADAMTS13 < 10%
		*n* = 15	*n* = 8	*n* = 2
Demographics	Female gender	5/15 (33%)	2/8 (25%)	2/2 (100%)
	Age at presentation (years)	14 (3–50)	15 (0.5–35)	57.5 (47–68)
Influenza type	A (non-H1N1)	7	–	2
	A(H1N1)	8	3	–
	B	–	4	–
	Undefined type	–	1	–
Renal status	Kidney transplant	2/15 (13%)	1/8 (13%)	0/2
Clinical aspects	CNS symptoms	8/15 (53%)	1/8 (13%)	1/2 (50%)
	Macrohematuria	6/14 (43%)	2/7 (29%)	0/2
Biological parameters^a^	Creatinine (μM)	327 (132–1,238), *n* = 11	309 (89–543), *n* = 8	462 (261; 650)
	Platelets (nadir)	21 (5–85), *n* = 14	25 (8–80), *n* = 8	6 (6; 6), *n* = 2
	Hemoglobin	77 (50–105), *n* = 14	92 (57–130), *n* = 7	108 (66; 150), *n* = 2
	LDH (U/L)	4,142 (847 ≥ 6,000), *n* = 8	2,810 (300–13,188), *n* = 8	2,100 (200; 4,200), *n* = 2
Complement and coagulation	C3 low	2/8 (25%)	3/7 (43%)	NR
	ADAMTS13 < 10%	0/2	0/2	2/2 (100%)
	FDP	5/7 (71%)	NR	1/1
Therapy	RRT (dialysis)	8/15 (53%)	1/8 (13%)	1/2 (50%)
	Plasma infusion	5/9 (44%)^b^	1/7 (14%)	0/2
	PLEX	6/9 (67%)^b^	4/7 (57%)	2/2 (100%)
	Anti-complement (eculizumab)	0/15	2/8 (25%)	0/2

*FDP* fibrin degradation products, * LDH* lactate dehydrogenase, *PLEX* plasma exchange, *RRT* renal replacement therapy

^a^Peak or nadir (or highest/lowest reported measurement)

^b^One patient was first treated with plasma infusion, followed by PLEX

^c^Seven patients with at least one pathogenic mutation (see Table 4); one patient (#13) with presumed membrane cofactor protein mutation (only tested for CFH, CFHR1, and anti-CFH antibodies)

## Pathogenesis of iHUS

There is an established link between influenza virus infection and HUS, but the underlying mechanism is speculative [45]. Influenza virus shares with *S. pneumoniae* the ability to produce neuraminidase. However, in vivo NA shedding by influenza virus is minimal (it is expressed on the viral membrane) compared with *S. pneumonia* [18, 50]. Its contribution to the pathogenesis of HUS has still to be shown.

Autopsy studies during the 2009 A(H1N1) pandemic revealed viral antigen in endothelial cells [51]. In vitro infection of endothelial cells by influenza virus [52] can trigger apoptosis [53], a process known to stimulate platelet adhesion directly and via the exposure of extracellular matrix [54, 55]. In addition to injuring or activating vascular endothelial cells, influenza virus may directly affect platelets. A(H3N2) virus induces clumping of human and rabbit platelets in vitro, and a rapid drop of platelet counts in vivo after injection of the virus into rabbits [56]. More recent studies have confirmed the potential of influenza virus to activate platelets and generate thrombin [57, 58]. In a prospective study comparing patients with acute respiratory distress syndrome (ARDS) due to severe influenza A(H1N1) and bacterial pneumonia with healthy controls, influenza showed the greatest degree of platelet activation measured as the formation of platelet-monocyte aggregates and activation of αIIbβ3 integrin on platelets [57].

## Influenza virus and complement

Complement regulates influenza virus-induced inflammation in the lung, enhances viral clearance, and protects against severe influenza infection [59]. Considered an important upstream mediator of the innate immune system, complement also bridges innate and adaptive immunity and is tightly linked to the coagulation cascade [60, 61]. More than 50 known complement proteins are expressed by hepatocytes, but also by tissue macrophages, blood monocytes, and renal and gastrointestinal epithelial cells [62]. Complement can be activated via three recognized pathways: alternative, classical, and mannan-binding lectin pathways, all resulting in the enzymatic cleavage of C5, the formation of the membrane attack complex (MAC) and of potent chemokines [61, 62]. It exerts both protective and potentially deleterious effects: it protects through virus neutralization via direct aggregation, opsonization, lysis, and promotion of phagocytosis involving complement receptors, and indirectly enhances T- and B-cell responses [61]. Complement also contributes to influenza-associated respiratory tissue injury, e.g., due to the generation of potent proinflammatory peptides [63, 64]. Neutralization of influenza virus in serum is mediated by the classical complement pathway via virus-reactive IgM [65]. Complement, complement receptors, and natural IgM antibodies appear to contribute to the maintenance of long-term memory of the influenza virus. However, influenza virus particles can activate complement in the absence of antibody [64].

Viral activation of the APC in the respiratory tract leads to ciliary dysfunction in vitro and increased levels of C3a and C5a in bronchial lavage fluid and serum of patients with severe influenza [66, 67]. Complement C5 activation during influenza A virus infection contributes to neutrophil recruitment and lung injury in mice [20]. Berdal et al. reported a > 10-fold increase in plasmatic levels of soluble MAC (sC5b-9) in patients with severe influenza by the pandemic A(H1N1) strain, indicating systemic complement activation [68].

Influenza-associated acute lung injury (ALI) in A(H5N1)-infected mice has been linked to excessive complement activation with deposition of C3 and C5b-9, and increased expression of complement receptors C3aR and C5aR. Treatment with a C3aR antagonist alleviated pulmonary inflammation in this model [69]. In another study, prevention of C5a release dampened inflammatory reactions caused by severe influenza A virus infection [63]. Treatment with anti-C5 antibody or C5a blockers inhibited influenza A virus-induced granulocyte activation and ALI. However, C3 and lytic MAC formation was protective in controlling murine influenza A virus infection [59]. The study confirmed earlier results showing delayed influenza virus clearance from the upper respiratory tract, reduced T-cell priming, and viral spreading to the lungs in C3-deficient mice [70].

## Complement abnormalities associated with iHUS

In our literature survey, plasma C3 levels were reported in 14 patients with iHUS; they were reduced in 5 (36%) and C4 was normal in 11 cases (Table 2). Eight patients, 4 with influenza A and 4 with influenza B infection respectively, underwent genetic screening. Seven were found to have AP component defects, including 3 patients with previous HUS episodes and a teenager with a renal allograft (Table 4). The latter patient carried a C3 gain-of-function mutation and had lost two previous kidney transplants owing to HUS recurrences; he was successfully treated with eculizumab for influenza-triggered HUS [33]. In addition, a 15-year-old girl (#13) was reported to have a suspected MCP mutation (based on the history of frequently relapsing aHUS that resolved spontaneously). The genetic workup was incomplete and showed normal C3 and C4, factor H, and factor I levels, undetectable CFH antibody, and lack of *CHF* mutation or CFHR1 deletion (Aysun Çaltik, personal communication). Interestingly, all four children with influenza B-associated HUS, published in 2017, carried one or more mutations of complement-related proteins [22, 23]. Although the number of genetically tested iTMA patients is small, we noted a high representation of C3 (3 out of 7) and MCP mutations (4 out of 7), combined with mutations of the clusterin and CFB genes respectively (Table 4).

**Table 4:** Complement and related gene mutations in patients with Influenza-associated TMA

Patient (age in years)	Influenza type	Previous episodes of HUS	MCP	C3	Other identified mutations	Plasma C3/anti-CFH	Other mutations tested	Treatment/outcome	Reference
#7 (17)	pA(H1N1)	Yes (1)	MCP splice acceptor site c.287-2A < G (IVS2-2A > G)^b^	–	–	Reduced/negative	CFH, CFI, C3, CFB normal	PLEX Complete remission	[26]
#13^a^ (15)	pA(H1N1)	Yes (4)	–	–	–	Negative	CFH, CFHR1 normal^c^	FP, then PLEX Complete remission	[30]
#16 (15)	pA(H1N1)	Yes (4) Lost 2 previous allografts to TMA Current 3rd allograft FHx of aHUS	–	C3 (ex. 14) 1835C > T R570W Gain-of-function	–	Reduced/ NR	NR^c^	PLEX, then eculizumab Stable graft function	[33]
#18 (35)	Influenza A	No	–	C3 c.3470 T>C p.I1157T	CFH low frequency variants of unknown significance c.3172 T>C (p.Y1058H) c.3178G>C (p.V1060 L)	NR/NR	CFI, CFB, THBD	NR	[43]
#22 (10)	Influenza B	First episode Family history of ESRD due to aHUS with low C3	–	C3 (ex. 4) c.481C > T p.Arg161Trp Gain-of-function	–	NR/NR	NR^c^	Recovery with “conservative” therapy	[22]
#23 (15)	Influenza B	Yes (3)	MCP (ex. 6) c.811-816delGACAGT p.Asp271-Ser272del	-	Clusterin (ex. 7) c.1298A > C p.Thr433Asn	NR/NR	NR	PLEX Complete recovery	[22]
#24 (9)	Influenza B	Yes (1)	MCP (ex.1) c.565 T > G p.Tyr189Asp	–	CFB (ex.1) c.26 T > A p.Leu9His Gain-of-function	NR/NR	NR	PLEX Complete recovery	[22]
#25 (0.5)	Influenza B	No, but early relapse during described episode	MCP c.104G > A p.Cys35Tyr	–	Non-allelic homologous recombination in RCA gene cluster on chr 1	Reduced/NR	CFH, CFI, C3 normal	Eculizumab Complete recovery	[23]

*CFB* complement factor B, *CFH* complement factor H, *CFHR1* CFH-related protein 1, *CFI* complement factor I, *ESRD* endstage renal disease, *MCP* membrane cofactor protein (CD46), *NR* not reported, *pA(H1N1)* pandemic A(H1N1), *PLEX* plasma exchange, *THBD* thrombomodulin

^a^Presumed MCP mutation, based on clinical course, but only tested for CFH mutation, CFHR1 deletion, and anti-CHF antibodies

^b^Splice acceptor site of intron 2

^c^No other test results were reported for these patients

Ten of the 25 reported patients with influenza-related TMA in our survey (40%) were involved in the 2009 A(H1N1) pandemic (Table 1). Some authors raised the question of whether the pandemic A(H1N1) strain poses an increased HUS risk [45]. This hypothesis is interesting in light of the case series by Berdal et al., who noted evidence for vigorous complement activation (and a tenfold increase in MAC levels in plasma) in patients with severe (complicated) influenza infection (none had HUS) [68].

We postulate that infections by microbial agents with potent complement-activating capacities, including certain influenza virus strains, confer an enhanced risk of inducing HUS in patients with APC regulator protein haplo-insufficiency. Data from various laboratories suggest a complex interplay between environmental factors (such as highly-active complement-activating biological agents) and risk haplotypes (combined mutations or risk polymorphisms) [1, 71], which may contribute to the variable, incomplete penetrance of genetic forms of aHUS.

## TMA following influenza vaccination

Thrombotic microangiopathy has been linked to influenza vaccines in a few adults since at least 1973 (median age 51 years, range 23–56 years). Analysis of five accessible reports showed a median interval of 2 weeks (4 days to 3 months) between immunization and onset of HUS (*n* = 2) and (presumed) TTP (associated with depleted ADAMTS13 activity and/or increased anti-ADAMTS13 antibodies; *n* = 3) [43, 72–75]. Direct and indirect Coombs tests were negative in 3 out of 3 patients, and 1 out of 2 patients demonstrated increased FDP levels. One patient tested negative for anti-CFH antibodies (#4), but no other complement studies were performed or reported (see Table 6). Disease manifestations and severity were highly variable: HUS cases (#1 and #5) were relatively mild, and both patients recovered with supportive treatment, with or without added prednisone [43, 72]. In contrast, TTP patients underwent prolonged PLEX, rituximab (#3 and 4) [74, 75], or vincristine treatment and splenectomy (#2) [73].

The pathomechanism linking TMA with influenza vaccines is poorly understood. The clinical phenotype and spectrum or TMA (HUS, TTP) following natural influenza infections and post-vaccination are comparable. However, none of the 5 patients required renal replacement therapy. As with iHUS and idiopathic TTP (iTTP), complement studies and screens for APC and related gene mutations are necessary for a rational treatment strategy. Similar to natural infections, flu vaccines may induce anti-ADAMTS13 antibodies [48] and activate complement directly and cause HUS in patients with certain risk haplotypes [71].

## Neuraminidase and the link between influenza and* Streptococcus pneumoniae* infections

*Streptococcus pneumoniae*, a Gram-positive, α-hemolytic, facultative anaerobic bacterium commonly colonizes the human nasopharynx. Commensal strains form biofilms without causing disease [76]. Pathogenic strains are responsible for invasive pneumococcal disease (IPD), including pneumonia, otitis media, meningitis, and peritonitis arising from the respiratory tract [77, 78].

Influenza virus is known to increase host susceptibility to (severe) *S. pneumonia* infection [79]. There is bi-directional interaction between these two pathogens [45, 79]. Neuraminidase (Nan) is an important virulence factor of pathogenic pneumococcal strains, supporting colonization and sepsis in vivo [80]. Ubiquitous NanA hydrolyzes α2,3-, α2,6-, and α2,8-sialyllactose to release *N*-acetyl-neuraminic acid (Neu5Ac) [50]. Viral and pneumococcal NAs possess distinct quaternary structures, but their active sites are similar and susceptible to neuraminidase inhibitors (NAIs), such as oseltamivir [81, 82].

The risk of pneumococcal pneumonia rises transiently by an estimated 100-fold following influenza [79, 83]. Importantly, IPD has also been associated with HUS (pneumococcal or pnHUS), mostly with pneumonia/pleural empyema or (pneumococcal) meningitis [78, 84].

HUS caused by *S. pneumoniae* infection was first described by Fischer et al. in 1971 [85]. It predominantly affects children <2 years of age and constitutes up to 5% of all pediatric cases of HUS [86]. *S. pneumoniae*-derived circulating Nan cleaves membrane sialic acid residues, unmasking a core disaccharide structure, Galβ1-3GalNAcα1, known as Thomsen–Friedenreich (TF) antigen, on red blood cells, platelets, and glomerular endothelial cells. One hypothesis states that preformed IgM binds to TF antigen and induces a cascade of events leading to HUS [77]. Alternatively, desialylation of membrane proteins may interfere with CFH binding and regulatory function, resulting in transiently unregulated APC activation as a cause of HUS. A recent study by Szilágyi et al. demonstrated signs of complement activation in all five described patients with pnHUS; three of them carried pathogenic mutations and potential risk haplotypes [87].

pnHUS patients are commonly Coombs test-positive, a feature that has been related to Nan-mediated desialylation [3, 84, 85]. By comparison, the direct Coombs test was negative in 7 of the examined iHUS patients (Table 2). Cold agglutinins were noted in a single case (#1; Table S1) [37], but their significance is unclear. Influenza virus produces quantitatively less NA than pneumococci. Viral NA is membrane-associated [18, 81], but may suffice to transiently disturb APC control. Of note, influenza virus-mediated desialylation of cell membrane glycans has been linked to vigorous C3b deposition and alternative pathway activation [88].

Influenza NA catalyzes cleavage of terminal sialic acid residues on epithelial membrane glycoproteins and glycolipids, providing mucin as a carbon source for rapidly increasing pneumococci leading to enhanced bacterial loads and severe infection/pneumonia [79, 89]. Similar metabiotic mechanisms have been postulated for influenza virus, and *H. influenzae* and *S. aureus* respectively, in addition to NA-producing parainfluenza virus [90]. We identified one case of influenza A iHUS that was complicated by *S. pneumoniae* infection and associated with unmasking of the TF antigen (case #8, Table S1) [41].

McCullers noted that treatment with NA inhibitors protects against secondary bacterial pneumonia, possibly because of medication-induced, reduced availability of viral NA [91]. It is intriguing to hypothesize that NA contributes to the growth of sialic acid-dependent pneumococci and alters complement resistance and APC regulation, including the binding of CFH on human tissue [24, 42]. Consequently, NA inhibitors may interfere with virus-induced complement dysregulation.

## Influenza TMA in kidney transplant recipients

Kidney biopsies, where obtained during acute and post-acute iHUS reveal varied features [25, 33, 37, 38, 92, 93] that replicate key findings described in other forms of HUS [3], including endothelial cell swelling and luminal narrowing, focal mesangiolysis, intravascular deposition of fibrin, and diffuse granular staining for C3 [25, 33, 37, 92, 93], and in some cases, for IgG or IgM [25, 37, 92]. No electron-dense deposits, virus-like particles or tubuloreticular inclusions were described [93]. The latter authors failed to demonstrate influenza A H3-specific hemagglutinin RNA using nested RT-PCR in the renal tissue [93].

## Laboratory diagnosis in patients with suspected iHUS

**Fig. 3:**
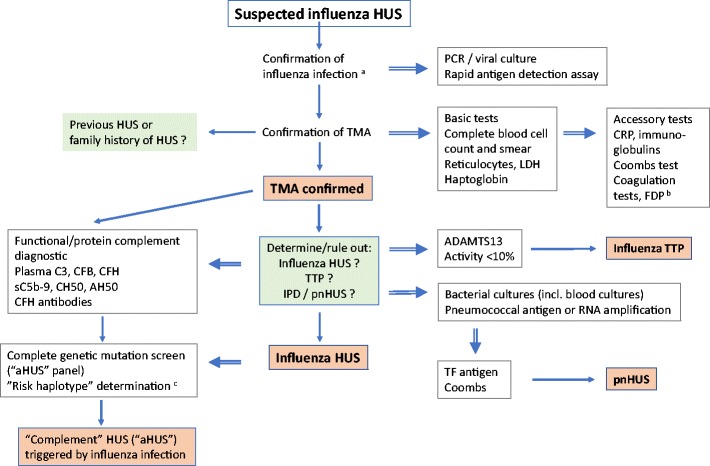
Diagnostic algorithm for influenza HUS and related thrombotic microangiopathies.* a* Influenza (or parainfluenza) virus;* b* the detection in plasma of fibrin/fibrinogen degradation products (such as d-dimers), but full-blown disseminated intravascular coagulation is not common and does not preclude the diagnosis of HUS;* c* combined complement regulator or coagulation protein mutations (e.g., membrane cofactor protein [MCP] and complement factor H (CFH) or single nucleotide polymorphisms (SNPs) in promoter regions [71]. *aHUS* atypical HUS, *CFB* complement factor B,* CRP* C-reactive protein, *FDP* fibrin/fibrinogen degradation products,* IPD* invasive pneumococcal disease,* LDH* lactate dehydrogenase, *pnHUS* pneumococcal/neuraminidase HUS,* PCR* polymer chain reaction, *TF antigen* Thomsen–Friedenreich antigen (Galβ1-3GalNAcα1), *TMA* thrombotic microangiopathy, *TTP* thrombotic thrombocytopenic purpura

All patients with HUS associated with seasonal or epidemic influenza should undergo rapid testing for ADAMTS13 activity, plasma C3, global hemolytic capacity (CH50 and AH50), circulating MAC (sC5b-9) and anti-CFH antibodies, in addition to genetic studies targeting HUS-associated complement and coagulation factors (Fig. 3). The detection in plasma of fibrin/fibrinogen degradation products (FDP; d-dimers), but not overt disseminated intravascular coagulation is common and does not preclude the diagnosis of HUS (Table 2) [29, 32, 36, 38, 39]. Blood samples for complement protein and functional measurements must be taken before initiation of plasma or anti-complement therapy [94]. TTP is suspected in patients with MAHA with very low platelet counts and mild renal injury [5]. Interestingly, the 2 patients in this survey with bona fide TTP had a substantial rise in serum creatinine and one of them was dialyzed (Table 3). The presence of complicating pneumococcal pneumonia or sepsis should be ruled out in any case of (suspected) iHUS using blood cultures, direct Coombs test, coagulation studies, rapid antigen detection or nucleic acid-based assays, and evidence for NA activity (Fig. 3) [78, 84].

## Therapeutic management, outcome and prevention

Patients with influenza-associated TMA benefit from best supportive care, similar to other forms of HUS [3, 84]. The outcome of iHUS is generally favorable; in our analysis, 3 out of 22 patients died (14%; #3, 4, and 9; all before 2008). Death was attributed to cardio-respiratory failure and/or CNS complications. All but one survivor recovered renal function after a median of 3 weeks (range 11–62 days; Table 2). In one of the kidney transplant recipients, active TMA only ceased after graft nephrectomy. Interestingly, the patient was successfully retransplanted without preventive measures (#1) [37]. There were no fatal outcomes or development of CKD during the A(H1N1) pandemic and the recent era (Table S1).

In comparison, STEC HUS is associated with mortality rates in children of <4% during the acute illness, irrespective of the infecting STEC serotype [78, 95], and about 20% develop generally minor, long-term renal dysfunction [78, 96]. Mortality rates of pnHUS vary between 2 and 12% (up to 37% in those with pneumococcal meningitis) [78, 86, 97, 98], and are comparable with those found in the current iHUS survey. Analyses of *S. pneumoniae-* and influenza-associated HUS series are confounded by small numbers and an unknown proportion of patients with complement regulator defects [22, 87].

The efficacy, tolerability, and safety of NA inhibitors for the prevention and management of influenza infections have been demonstrated in large clinical trials that also included infants [99]. Flu vaccination reduces the incidence of pneumococcal HUS [78]. Oseltamivir also improved the outcome of secondary pneumonia, and subsequent treatment with an antibiotic led to 100% survival in a murine influenza infection model [91]. However, there is no experimental model of iHUS or iHUS/TTP prevention, and current evidence is lacking as to whether NA inhibitors prevent or ameliorate influenza TMA. In our analysis, 71% of treated and reported patients received the NA inhibitor only after the diagnosis of HUS had been made (Table 2).

Individual and population immunity against endemic or epidemic influenza strains is expected to reduce the occurrence of iTMA. However, the immunization history is rarely mentioned in the available case reports. Furthermore, the notorious variability of the predicted antigen changes hampers the efficacy of influenza A vaccines [17]. Although vaccination has been linked to HUS or TTP in a few instances (Table 5), data are scarce and should not be construed as an argument against active immunization. In contrast to natural infections, vaccination allows monitoring for signs of post-vaccination TMA and prompt intervention in persons with a history of (atypical) HUS.

**Table 5:** Influenza vaccine-associated TMA: review of accessible publications

Number	Case	Influenza vaccine	Diagnosis (history)	Clinic presentation	Laboratory parameters	Coombs FDP	ADAMTS13	Complement	Treatment	Outcome	Reference
1	23 years, female, UK	Influenza vaccine^a^	TMA/ HUS?	14 days after vaccination, bruises, after further 7 days pallor BP 180/95 mmHg	Hb 92 g/L Plt 39/nL Urea 15.9 mM (34 mg/dL) Schisto +	Coombs negative, fibrinogen, FDP within normal range	NR	NR	PRBC, Plt transfusions Heparin Prednisolone	Recovery 1 week after starting prednisolone	[72]
2	51 years, male, 2000, France	Influenza vaccine^b^	Relapsing TTP (after influenza vaccine)	TTP relapse #1 three months after vaccination, relapse # 2 two months after second vaccine	*Episode 1*:Hb 100 g/LPlt 10/nLLDH 1,050 U/LSchisto ++*Episode 2*: Plt 30/nL LDH 1,060 U/L	Coombs negative (at time of TTP diagnosis)	ADAMTS 13 < 5% Inhibitor high (both at the time of the second relapse)	NR	PLEX against FP (6 and 5 sessions, respectively) VCR splenectomy during preceding episodes of TTP, prior to Influenza vaccination	Recovery after 6 and 5 PLEX, respectively	[73]
3	54 years, male, UK	Influenza vaccine ^c^	Presumed TTP (Hx of T2DM, HTN, MI)	4 days after vaccination CNS: agitation, confusion, digital ischemia (hand)	Hb 57 g/L Plt 7/nL Cr 134 μM LDH 4,183 IU/L Schisto +	D-dimers 6,258 ng/mL (0–210)	(Samples sent after first PLEX) ADAMTS 13 21% Anti-ADAMTS13 IgG positive	NR	PLEX (21 days) Mechanical ventilation Rituximab	Recovery after 29 days	[74]
4	56 years, male, Germany	A(H1N1) vaccine^d^	Presumed TTP	13 days after vaccination petechiae, CNS: confusion, frequent seizures	Hb 45 g/L Plt 17/nL Cr 116 μM LDH 40.53 μmol/L/s (*n* < 4.13) Schisto 24% (*n* < 0.5%)	Coombs negative	ADAMTS13 67% Ag 0.06 μg/mL (*n* 0.50–1.60) Inhibitor >111 U/mL (*n* < 16) VWF multimers +	Anti-CFH Ab negative No other complement studies reported	MPred pulses PLEX against FP (>46) Rituximab (4 doses)	Subacute bilateral infarction of basal ganglia Recovery (>5 weeks)	[75]
5	38 years, female, Japan	NR	HUS (first TMA at age 21 years)	Interval not reported, AKI, CNS involvement	Hb 98 g/L Plt 35/nL Cr 124 μM LDH 928 U/L	NR	92.5%	NR	Supportive, no dialysis	Survived	[43]

*Ab* antibody, *Cr* serum creatinine, *FDP* fibrin/fibrinogen degradation product(s), *FP* fresh (frozen) plasma, *Hb* hemoglobin, *HTN* arterial hypertension, *LDH* lactate dehydrogenase, *MI* myocardial infarction, *NR* not ready, *PLEX* plasma exchange, *Plt* platelets,* PRBC* packed red blood cells, *T2DM* type 2 diabetes mellitus,* VCR* vincristine, *VWF* von Willebrand factor

^a^Flenzavax (influenza A split vaccine treated with sodium deoxycholate after formaldehyde)

^b^Fluvirin, Celltech Pharma (trivalent, inactivated subunit influenza vaccine); Agrippal, Socopharm (trivalent [A and B] inactivated surface antigen vaccine)

^c^Inactivated influenza vaccine (split virion) BP, Sanofi Pasteur MSD

^d^Pandemrix®, GlaxoSmithKline (monovalent split A(H1N1) immunological adjuvant AS03-enhanced vaccine [100]

## Is there a role for plasma infusion, PLEX or anti-complement agents in iHUS?

Our survey covers a period of 46 years representing different eras, seasonal and epidemic influenza strains (Table 1), and therapeutic approaches (Tables 2, S1). Overall, 16 out of 24 patients with available data received any plasma therapy (67%): 5 were treated with plasma infusions and 12 were treated with PLEX (1 patient received both modalities; Table 2). Two patients were given eculizumab: patient #16, carrier of a C3 gain-of-function mutation, who had lost two previous renal allografts owing to recurrent HUS [33], received eculizumab when HUS recurred in the third allograft during the A(H1N1) influenza pandemic. Patient #25 was a 6-month-old infant with influenza B-associated HUS who received eculizumab when he relapsed while recovering from his first episode of aHUS. He was later shown to have a heterozygous MCP mutation (Table 4) [23].

Treatment of iHUS hinges on the direct effects of the influenza infection, complicating bacterial pneumonia, “best” supportive care, including dialysis and blood products, if needed, and plasma or anti-complement therapy (e.g., eculizumab), particularly in the presence of complement regulator deficiencies (Table 6). Recommendations for “atypical” HUS, including anti-complement agents or PLEX, should be implemented in patients with iHUS who present functional or genetic evidence for complement dysregulation or have a previous history of (atypical) HUS, a positive family history of (a)HUS, or HUS recurrence after kidney transplantation (Table 6). In the case of profound ADAMTS13 depletion and suspected TTP, most authors recommend PLEX and immunosuppressive therapy [2, 84, 94].

**Table 6:** Treatment of influenza-associated TMA

Treatment	Details	Comments/references
Best supportive care	Respiratory support	
	Intravascular volume status	
	Blood pressure control	
	Blood products (PRBC, platelet transfusion)	
	Diuretics	Only after sufficient intravascular volume
Renal replacement therapy	HD, PD, CRRT	Based on tolerability, circulatory, and cardiac stability Expertise and equipment availability
Antimicrobial therapy	NA inhibitors (e.g., oseltamivir)	Potential to prevent HUS if given early during infection (or at exposure?) [97]. Preventive efficacy remains to be proven
	Antibiotics (3rd generation cephalosporins and others)	Antibiotics reduce rates of complicating bacterial pneumonia and possibly pnHUS [79, 89]
Plasma and anti-complement therapy	Plasma exchange (PLEX) (Plasma infusion, PI)	Option for patients with complement dysregulation and/or evidence of autoimmune TMA/TTP (anti-CFH or anti-ADAMTS13) PI restricted to unavailability of PLEX Note: spontaneous recovery of iHUS may occur (see Tables 2 and S1)
	Anti-complement antibody	Treatment of choice for children with iHUS and suspected or proven complement dysregulation (pathogenic mutation, relapsing/recurrent HUS) [23, 33]

*CRRT* continuous renal replacement therapy, *HD* hemodialysis, *NA* neuraminidase, *PD* peritoneal dialysis, *pnHUS* pneumococcal/neuraminidase-associated HUS, *PRBC* packed red blood cells, *TMA* thrombotic microangiopathy, *TTP* thrombotic thrombocytopenia

## Conclusions and future areas of research

Influenza-associated HUS or TMA is rare. It has been linked to influenza A and influenza B infections and, in several instances, pathogenic complement gene mutations. The outcome is generally favorable, but depends on underlying complement gene deficiencies and/or the presence of CFH or ADAMTS13 autoantibodies. Identification of the etiology and differentiation between HUS due to complement dysregulation, where the Influenza virus may act as a potent trigger, and influenza-associated HUS without identifiable complement abnormalities, are critical for acute and long-term management.

The term “atypical” HUS has been originally coined to describe a heterogeneous group of infrequent forms of HUS not associated with STEC colitis. The current, interchangeable use of the epithet “atypical” that may or may not include HUS owing to pneumococcal infection and various metabolic and other conditions, including “secondary” forms of HUS [1, 7] and HUS strictly caused by complement dysregulation (“primary complement-mediated HUS” or “HUS with dysregulation of the APC” [2]), leads to confusion about the etiology of and appropriate therapy for different forms of HUS. We argue in favor of etiologically defined designations that correspond to different pathomechanisms and lead to rational, mechanism-targeting therapies [2, 3, 94].

As detailed in this review, the pathogenesis of influenza virus-induced TMA is varied. It is intriguing to hypothesize that influenza-derived NA plays a causative role. Membrane glycan desialylation by functionally active, virus membrane-bound NA may cause transient loss of resistance to APC activation on epithelial and/or vascular endothelial cells. Alternatively, virus-specific, alternative mechanisms of (microvascular) endothelial injury resulting in an HUS phenotype need be explored. Both warrant further studies.

The diagnostic workup in most of the reviewed cases is incomplete from today’s perspective. Some influenza strains appear to be potent alternative pathway activators in vivo [68] and therefore trigger HUS in non-immune individuals, uncovering specific regulator haplo-insufficiency or changes in noncoding complement gene sequences [71]. Although the number of genetically studied iHUS cases is small, it is noticeable that all identified patients carried mutations in the MCP or C3 gene, occasionally combined with other mutations. Further delineation of “risk haplotypes” and specific microbial agents or their products could have therapeutic and preventive implications and will advance our understanding of this intriguing disease group.

At present, general treatment recommendations for iHUS and iTTP are lacking. In view of the high proportion of patients with APC dysregulation, PLEX or anti-complement agents constitute a reasonable therapeutic approach, while striving for a rapid and comprehensive etiological diagnosis.

## Electronic supplementary material

ESM 1 (DOCX 151 kb)

## Abbreviations

ADAMTS13: A disintegrin-like metalloproteinase with thrombospondin type 1 motif 13
aHUS: Atypical hemolytic uremic syndrome
AKI: Acute kidney disease
APC: Alternative pathway of complement
CFB: Complement factor B
CFH: Complement factor H
CFHR: Complement factor H-related protein
CFI: Complement factor I
CKD: Chronic kidney disease
CNS: Central nervous system
DCT: Direct Coombs test (direct agglutination test)
DD: Deceased (kidney) donor
DGKE: Diacylglycerol kinase-epsilon
ESRD: End-stage renal disease
FDP: Fibrin/fibrinogen degradation products
FP: Frozen plasma
HA: Hemagglutinin
Hb: Hemoglobin
HD: Hemodialysis
HUS: Hemolytic uremic syndrome
iHUS: Influenza-associated HUS
IPD: Invasive pneumococcal disease
iTMA: Influenza-associated thrombotic microangiopathy
KT: Kidney transplant(ation)
LDH: Lactate dehydrogenase
MAC: Membrane attack complex (C5b-9)
MAHA: Microangiopathic hemolytic anemia
MCP: Membrane cofactor protein (CD46)
NA: Neuraminidase (influenza)
Nan: (Pneumococcal) neuraminidase
PD: Peritoneal dialysis
PI: Plasma infusion
PLEX: Plasma exchange
PLG: Plasminogen
Plt: Platelet(s)
pnHUS: Pneumococcal/neuraminidase HUS
PRBC: Packed red blood cells
sC5b-9: Soluble (vitronectin-bound, plasmatic) membrane attack complex
STEC: Shiga toxin-producing *Escherichia coli*
TF Ag: Thomsen–Friedenreich antigen
THBD: Thrombomodulin (CD141)
TMA: Thrombotic microangiopathy
TTP: Thrombotic thrombocytopenic purpura
VWF: Von Willebrand factor
