# Supplementary material for: Influenza-associated thrombotic microangiopathies
Source: Pediatr Nephrol. 2017 Sep 7;33(11):2009–25. doi: 10.1007/s00467-017-3783-4 (PMC6153504; doi:10.1007/s00467-017-3783-4)
Supplement: Supplementary file 1 — (DOCX 151 kb) [file 467_2017_3783_MOESM1_ESM.docx]

**Supplementary Table S1**. Detailed account of identified influenza TMA patients (HUS or TTP) ^a^

| **#** ^a^ | **Demographics**  (season, country) | **Influenza serotype and diagnostic** | **Underlying disorder** | **HUS (TMA) presentation**  Time after onset of influenza | **Labs**  Hematology, coagulation, creatinine (peak/nadir)  Proteinuria (Up) | **Coombs test**  **Coagulation** | **Complement ADAMTS13** | **Kidney Biopsy** | **Treatment** | **Outcome** |
| --- | --- | --- | --- | --- | --- | --- | --- | --- | --- | --- |
| 1 | 20 y F  Jan 1970  Denmark  [[36](#_ENREF_36)] | Influenza A (serology) | ESRD due to acute proliferative GN,  malignant hypertension  KT | 2 weeks of fever, fatigue  (23 mo post KT) | HCT 0.37🡪0.172, reticulocytosis  Plt 56/nL  Cr 230 🡪1238 µM  Up 5 g/L | Coombs neg  Cold agglutinins pos 1:64 (peak) | C3 “below normal”  (normalized after graft removal)  ADAMTS13 N/R | 5 weeks of onset Thrombosis of small arteries/glom capillaries. IF IgG, IgM, fibrin (weak C3 and IgA) | HD ~10 d prior to transplant nephrectomy  Graft removal 8 weeks after HUS onset | Immediate Plt normalization post KT Nx Successful 2^nd^ KT |
| 2 | 14 y F  -  UK [[37](#_ENREF_37)] | Influenza A (serology)  Bacterial cultures neg | Previously healthy | 2 days  Gross hematuria, hemoptysis, drowsiness, jaundice, oliguric AKI | Hb 78 g/L  Plt 30/nL  Schisto +  Urea 111 mM  Up N/R | DIC  Fibrin degradation | N/R | Bx 1 - Day 4 of illness & at recovery  Diffuse endothelial and mesangial hypercellularity  IF fibrin +, focal ATN  Bx 2 - Resolution of findings | HD (5 sessions) | Full recovery |
| 3 | 14 y M  -  UK [[37](#_ENREF_37)] | Influenza A culture (trachea, lung)  Blood culture negative | Previously healthy | 4 days  Massive hemoptysis | Hb 105 g/L  Plt <10/nL  Schisto +  Urea 22 mM  Na 124 mM  Up N/R | Fibrin degradation | N/R | Autopsy  Mesangial hypercellularity, denuded endothelium (brain petechial hemorrhage) | Fulminant course | Death from massive hemoptysis on day of admission |
| 4 | 50 y M  December 1977  USA [[89](#_ENREF_89)] | Influenza A2  Throat culture | Previously 2 episodes of transient aphasia and obtundation after flu-like illnesses 1974 & 1975 w/o TMA  Previous KBx chronic GN w IgG and C4 deposits and apparent GBM duplication | 3 days  Severe MAHA, focal neurological signs, confusion | Hemolytic anemia, schisto +  Profound thrombo-cytopenia  Cr N/R  Up 11.2 g/d | Normal fibrin split products | N/R | Glom capillary fibrin thrombi, RBC fragments, endothelial swelling Reduplication of GBM  IF: granular capillary fibrin, C3 & IgM; mesangial IgG & fibrin deposits | PLEX  Prednisone  Whole blood transfusion  Anti-Plt agents  splenectomy | Protracted disease course, death from aspiration pneumonia |
| 5 | 34 y M  January 1997  Japan [[90](#_ENREF_90)] | A(H3N2) (serology)  HA Ab 32🡪256 | ESRD due to chronic GN  KT | 4 days (?)  (1 y post KT)  Dark urine, HTN | HCT 0.298  Plt 15/nL  Schisto +  LDH 2888 U/L  Hp undetectable  Cr 212 🡪 327 μM  Up 4.1 g/d | Coombs neg | C3/C4 normal | Day 10: Glomerular endothelial cell swelling and focal  mesangiolysis.  IF pos C3, IgM glom cap walls, no deposits by EM  No evidence of graft rejection | FP infusions (5 d) anticoagulation  MPred pulses | Graft function recovery |
| 6 | 3 y F  March 1998  Japan [[91](#_ENREF_91)] | A(H3N2) (throat swab culture, serology)  Bacterial pathogens negative  HA inhibition 1:1,024 | Previous history N/R | 5 days  High fever, pale, petechiae, edema;  Oliguric AKI  seizures  cardiomegaly | Hb 65 g/L  Plt 53/nL  LDH 2316 U/L, Hp indetectable  Cr 318 μM  Up N/R | Coombs neg  Plasma aPTT, TM and TNF-α ↑ Normal IL-1β, IL-6, INF-γ  Normal fibrinogen & FDP | N/R | Day 30: mesangial hypercell & matrix, no fibrin thrombi  IF: C3 pos  EM: no deposits or viral material | HD (duration N/R) | Gradual recovery of renal function |
| 7 | 17 y M  October 2009  Portugal  [[24](#_ENREF_24)] | pA(H1N1) PCR nasal swab  Urine pneumococcal Ag neg | Previous HUS episode @ age 3 y (complete recovery) | Fever, jaundice, HTN 160/95 mmHg | Hb 72 g/L  Plt 8/nL  Schisto +  LDH 13,188 U/L  Cr 543 µM  Up N/R | Coombs neg  Coag normal | **MCP mutation**  C3 low 0.52,  C4 0.05 g/L,  Anti-CFH Ab neg  CFH, CFI, CFB normal  ADAMTS13 normal | N/R | PLEX 10, HD 2 sessions  prednisolone  oseltamivir  antibiotics | Complete recovery 3 weeks |
| 8 | 3.7 y F  -  Taiwan  [[87](#_ENREF_87)] | Influenza A & *S. pneumoniae* | none | 7 days  oliguria, anuria  decreased consciousness, brain MRI changes (d 23 of diagnosis of HUS) | Hb 123🡪86 g/L, Schisto +, LDH 2385 U/L  Plt 13/nL  Cr 156 µM  Up N/R  TF Ag pos | N/R  TF ag pos | N/R | no | HD 33 days  Frequent PRBC and Plt  Ventilation 17 d  CVVA, PD (33 d) | Normal renal function D62  Mild proteinuria |
| 9 | 68 y F  March 2007  Japan [[38](#_ENREF_38)]  *Bona fide TTP* | Influenza A from throat swab (immune-chromato-graphy) | Gastric cancer and uterine myoma surgeries @ age 47/48 y, otherwise healthy | 3 days  Generalized seizures, pulmonary and cardiac complications | Plt 6/nL  Hb 66 g/L LDH 3060 U/mL, schisto +  Cr 261 µM  Up N/R  TNA-α, IL-6, IL8 & CRP ↑ | ↑ of d-dimers 23.14 μg/mL (N<1), thrombin-antithrombin complex, and others coag abnormalities | C N/R,  **ADAMTS13**  **<0.5%,**  IgG inhibitor 6 U/mL (N < 0.5)  ADAMTS13 Ag 0.1 % | N/R | PLEX, MPred  Oseltamivir (D2 of influenza)  PRBC 840 mL total | Death pulmonary congestion/myocardial infarction after 3 d of PLEX |
| 10 | 37 y M  -  Portugal  [[25](#_ENREF_25)] | pA(H1N1)  PCR respiratory specimen | Previously healthy | Fatigue, dark urine, jaundiced skin and sclerae  HTN | Hb 74 g/L  Schisto +  5376 U/L  Plt 85/nL  Cr 336 μM  Up 3+  ALT normal | Coombs neg  Coag “normal”  FDG NR | C3, C4 normal  ADAMTS13  (0.33 μg/mL; normal range = 0.60–1.60 μg/mL) | No biopsy | PLEX (5 days)  No dialysis  PRBC and Plt transfusion NR (likely not given)  Oseltamivir started 2 post diagnosis of HUS | Full recovery day 15 of HUS |
| 11 | 7 y M  November 2009  Greece [[26](#_ENREF_26)] | pA(H1N1)  PCR respiratory specimen | Previously healthy | 5 days  Pneumonitis and pleural effusion,  HTN/PRES with generalized  seizures | Hb 62 g/L  Schisto +  Plt 12/nL  Cr 301 μM  Up N/R | Coombs neg  Coag and fibrinogen normal | C3, MCP normal  CFB, CFH, CFI normal (after recovery)  ADAMTS13 activity normal | Not reported | Plasma infusion,  PRBC, Plt transfusions  Oseltamivir  Abx  PD  ventilation | Improved on day 14,  complete renal recovery |
| 12 | 5 y F  -  USA [[27](#_ENREF_27)] | pA(H1N1)  RAD, PCR  Blood culture, endotracheal negative for *S. pneumoniae* | Healthy (one episode of pneumonia) | 6 days  Oral bleed, gross hematuria  Oliguric AKI | Hct 0.24  Plt 27/nL  Cr 24 🡪 247 μM  LDH N/R  Up 5 g/L | Coag profile normal  D-dimer ++  Fibrinogen normal  Coombs N/R | N/R | No biopsy | CVVHD/intermittent HD  PRBC, Plt transfusions  Oseltamivir D5 of influenza  Abx  CPAP | Anuria 15 days, dialysis 4 weeks  Complete recovery |
| 13 | 15 y F  December 2009  Turkey [[28](#_ENREF_28)] | pA(H1N1) (PCR) NP swab | Relapsing HUS  (preceding episodes at age 4, 12, 13, 14) | “few days”  5^th^ HUS episode  HTN 150/100 mmHg | Hb < 93 g/L  Plt < 48/nL  LDH 847 U/L  Schisto +  Cr 451 μM  Up 2+ | Coombs neg  Coag PT, aPTT normal | C3 0.979, C4 0.256 g/L normal  CFH, CHI levels normal  Anti-CFH neg,  No CFH mutation | No biopsy | PRBC, Plt, frozen plasma  No dialysis  oseltamivir | Complete recovery within 2 weeks |
| 14 | 27 y M  December 2010  Korea [[29](#_ENREF_29)] | pA(H1N1) (PCR, RAD)  Late complicating pneumonia (*S. aureus*, *A. baumannii*) | Previously healthy | 3 days  Alveolar hemorrhage, gross hematuria (later anuric)  normotensive | Hb 68 g/L  Plt 26/nL  LDH >6000 U/L, schisto +  Cr 283 🡪 701 μM  Up 2.52 g/g | Coombs neg | C3 0.699, C4 0.122 g/L  ADMAMT13 N/R | No biopsy | PLEX (17 sessions),  CVVHDF (12 days)  Glucocort for hemorrhage  Oseltamivir  Ventilation (until D29) | Complete recovery after about 6 weeks |
| 15 | 11 y M  -  Serbia [[30](#_ENREF_30)] | pA(H1N1)  *S. pneumoniae* neg (sputum, BCx) | Previously healthy | Interstitial pneumonia, jaundice, oliguric AKI | Hb 50 g/L  Plt 38/nL  Schisto +  LDH 4,800 U/L  Cr 280 μM  Up 3+  U RBC casts | Coombs neg  D-dimers +  Coag otherwise normal | C3/C4 normal | No biopsy | FP (1 d) 🡪 stopped b/o worsening hemolysis  No dialysis  Dopamine  Oseltamivir  Abx | gradual recovery over 15 d |
| 16 | 15 y M  -  USA [[31](#_ENREF_31)] | pA(H1N1)  Diagnostic method N/R | Recurrent aHUS  ESRD at age 16 mo  2 allografts lost to recurrence of HUS | 7 d  Gradual worsening, oliguric AKI  HTN  Current episode 8 weeks post 3^rd^ KT | Mild thrombocytopenia, mild LDH increase,  no schisto  Cr 186 🡪 256 μM  Up 2+ | N/R | **C3 mutation**  C3 persistently low, reduced 0.23 g/L during current HUS, C4 not reported | TMA  w narrowing of glom capillary  lumina and thickening of the capillary loops, mesangial  expansion, focal arteriolar C3 deposition | PLEX 10 sessions, partial response, then eculizumab (chronic)  Oseltamivir prior to HUS | Stabilized graft function and BP;  TMA resolved 6-12 mo |
| 17 | 12 y M  -  Greece [[32](#_ENREF_32)] | pA(H1N1)  PCR (NPA) | Previously healthy | 1 day  Presenting ecchymoses  On D12 hemiplegia and MAHA | Hb 95 g/L  Plt 5.060/nL  Schisto +  Retic 10%  Cr 132 μM  Up N/R | DCT and coag N/R | C and  ADAMTS13  N/R | No biopsy | PLEX x 30 d  No dialysis  oseltamivir | Complete recovery |
| 18 | 35 y F  -  Japan  [[71](#_ENREF_71)] | Influenza A (not defined) | NR | NR | LDH 4485  Cr 416 μM  Up N/R | NR | -  ADAMTS13 88.8% | No biopsy | Not detailed | favorable |
| 19 | 27 y M  December 2010  Korea [[33](#_ENREF_33)] | pA(H1N1)  RAD test, PCR sputum aspirate | NR | Fever, nausea, HA, pneumonia, mental confusion  normotensive  6 d | Hb 90 g/L  Plt 26/nL  Schisto 3+  LDH 6000 U/L  Cr 698 μM  Up N/R | DCT neg  PT, aPTT normal | C and  ADAMTS13 N/R | No biopsy | PLEX against FP and albumin (17 sessions)  MPred pulses  PRBC (total 7,920 mL) | Clinically normal after 17 PLEX  Complete recovery D47 of admission |
| 20 | 11 y M  -  Japan  [[34](#_ENREF_34)] | A(H1N1)  Genotyping  BCx neg | No previous Hx reported  Protein S haplo insufficiency (?) | 3 d  conjunctival jaundice, fever, no bleeding  reddish-brown urine | Hb 132🡪76 g/L  Plt 14🡪 /nL  LDH 3484 U/L  Schisto +  Cr 118🡪272 μM  CK 1228 U/L (N <249)  CRP 60 mg/L (N<2)  Ferritin 8999 μg/L (39-265)  Myoglobin 330 μg/L (0-60)  Urine blood 3+  Up 3+  U myoglob 4,932 μg/L (0-10) | Coombs NR  D-Dimer 2.19 (N < 0.01) mg/L  FDP 3.81 (N <0.05) mg/L  Fibrinogen N  INR & aPTT elevated | No C studies  ADAMTS13:Ac 68%  No inhibitors (<0.5 Bethesda units) | No biopsy | FP (1) mL  PLEX (3 ?)  MPred pulses  rTM, AT, inhibitors of trypsin (ulinastatin), & serine protease (nafamostat)  Zanamivir | Bilat PE  Recovery Plt and Cr 11 d, full recovery 28 d |
| 21 | 47 y F  -  France (Turkish origin)  [[42](#_ENREF_42)]    *Bona fide TTP* | immunochromatography on NP secretions | Asthma since childhood, no previous TMA | 2 days (estimated)  Tachycardia, tachypnea  Post-influenza pneumonia day 6 of HUS | Hb 150 g/L 🡪 N/R  Plt 57🡪6/nL  Schisto 3+  LDH 200 U/L  Cr 65🡪650 μM  Up N/R | Coombs NR  ANA and APL Ab neg | AH50 “normal”  **ADAMTS13 <5%,**  anti-ADAMTS13 high (70 IU/L) | No biopsy | PLEX (12)  HD (6)  PRBC (4)  Plt (0)  Oseltamivir at diagnosis of HUS  Pred 1mg/kg/d  Abx (cefotaxime, linezolide)  Early relapse of MAHA 🡪RTX, restarted Pred | Recovery after 11 or 28 days |
| 22 | 10 y M  Winter 2012/13  Belgium  [[22](#_ENREF_22)] | Influenza B (B/Yamagata lineage) NPS-RAD | Previously healthy | 3 days of flu-like symptoms  Macrohematuria, jaundice, petechiae, edema;  drowsiness | Hb 113 g/L  Plt 25/nL  LDH 2920 U/mL  Schisto +  Cr 135 🡪 182 μM  Up 12.58 g/g | N/R | C3 mutation  C3d 8.4% (N < 2.4%)  Normal C3, C4, CFB levels  ADAMTS13 normal | No biopsy | PRBC & platelet transfusions  No plasma therapy or dialysis | Complete recovery after 1 mo |
| 23 | 15 y M  Winter 2012/13  Belgium  [[22](#_ENREF_22)] | Influenza B (B/Yamagata)  NPS-RAD | Relapsing HUS with two preceding episodes | 2 days of flu-like symptoms  HTN  HTN, edema, oliguric AKI | Hb 13 g/dL  Plt 20/nL  LDH 5218 U/mL  Schisto +  Cr 362 μM  Up 4.08 g/g | N/R | MCP & clusterin mutations  C3d 10.1%  Normal C3, C4, CFB levels  ADAMTS13  normal | No biopsy | PLEX (n=6)  No dialysis  PRBC & Plt transfusions N/R | Gradual recovery, normalization of serum creatinine after 2 months |
| 24 | 9 y M  Winter 2012/13  Belgium  [[22](#_ENREF_22)] | Influenza B  NP swab, rapid antigen detection | Previous episode of HUS after *S. typhimurium* infection | 2 days of flu-like symptoms  with epistaxis, macrohematuria  Petechiae, jaundice | Hb 104 g/L  Plt 22/nL  LDH 2700 U/mL  Schisto +  Cr 89 μM  Up 4.76 g/g | N/R | MCP & CFB mutations  (MCP c.565T>G [p.Tyr189Asp]) (CFB c.26T>A[p.Leu9His] in exon 1, gain of function)  Normal C3, C4, CFB levels  ADAMTS13 normal | No biopsy | PLEX (6 sessions)  No dialysis | Full recovery, after 1 month |
| 25 | 0.5 y M  -  Germany  (Turkish origin, non-consanguinous)  [[23](#_ENREF_23)] | B/Yamagata | early relapse after described episode | 5 d  HTN  Proteinuria  oliguria | Hb 57 g/L  Plt 58 🡪25/nL  Schisto +  LDH peak 2150U/L  Cr 126 🡪 159 μM  Up “proteinuria” | C3 0.58 g/L  sC5b-9 450 ng/mL (N <320) | MCP mutation  ADAMTS13 normal | No biopsy | No dialysis  No plasma therapy  Eculizumab (ongoing)  Oseltamivir |  |

^a^ In order of publication dates (or acceptance for publication). Shaded fields = kidney transplant recipient; mutations are highlighted in red

Abbreviations: *Abx* antibiotics, *ADAMTS13* A disintegrin-like metalloproteinase with thrombospondin type 1 motifs 13, *CK* creatine kinase, *Cr* serum creatinine, *CRP* C-reactive protein, *d* days, *DCT* Direct Coombs test (direct agglutination test), *ESRD* endstage renal disease, *FDP* Fibrin degradation products, *FP* frozen plasma, *Hb* hemoglobin, *HD* hemodialysis, *HTN* hypertension, *MPred* methylprednisolone, *NPA* nasopharyngeal aspirate, *NPS* nasopharyngeal swab, *Nx* nephrectomy, *PD* peritoneal dialysis, *PLEX* plasma exchange, *Plt* platelets, *Pred* prednisone therapy, *Nx* nephrectomy, *RAD* rapid antigen detection, *rTM* recombinant TM, *RTX* rituximab, *TM* thrombomodulin, *Up* urine protein, *y* year(s)

SI conversions: Serum creatinine 1 mg/dL = 88.4 μM, Hb 1g/dL = 10 g/L
